# Cost-effectiveness analysis of tislelizumab plus chemotherapy as first-line treatment for HER2-negative advanced gastric or gastro-oesophageal junction adenocarcinoma

**DOI:** 10.3389/fphar.2025.1500729

**Published:** 2025-05-22

**Authors:** Liangliang Zou, Guilan Guo, Yufan Huang, Cuihua Yuan

**Affiliations:** ^1^ Mindong Hospital Affiliated to Fujian Medical University, Ningde, Fujian, China; ^2^ Department of Pharmacy, Shanghai Eastern Hepatobiliary Surgery Hospital, Shanghai, China

**Keywords:** tislelizumab, chemotherapy, cost-effectiveness, first-line treatment, HER2-negative, gastric or gastroesophageal adenocarcinoma

## Abstract

**Background:**

The RATIONALE-305 trial indicates that tislelizumab plus chemotherapy (TLE-CHM) offers clinical benefits over placebo plus chemotherapy (PLB-CHM) as a first-line treatment for patients with HER2-negative advanced gastric or gastro-oesophageal junction (G/GEJ) adenocarcinoma. Nonetheless, incorporating tislelizumab results in higher treatment costs, raising concerns about its cost-effectiveness relative to PLB-CHM. This study aimed to assess the cost-effectiveness of TLE-CHM as an initial treatment for HER2-negative advanced G/GEJ adenocarcinoma from the perspective of the Chinese healthcare system.

**Methods:**

A Markov partitioned survival model incorporating three health states was developed to evaluate the cost-effectiveness of TLE-CHM as a first-line treatment for advanced G/GEJ adenocarcinoma. Clinical data were sourced from the RATIONALE-305 trial, with drug costs calculated at the national tender price, and additional costs and utility values derived from published literature. The outcomes measured included total costs, quality-adjusted life years (QALYs), and incremental cost-effectiveness ratios (ICERs). Sensitivity analyses were conducted to validate the model’s robustness.

**Results:**

TLE-CHM achieved 1.53 QALYs at a cost of $23,484.39, compared to 1.14 QALYs at $12,123.52 for PLB-CHM. The ICER for TLE-CHM versus PLB-CHM was $29,608.51 per QALY gained. Key parameters influencing the model results included PFS utility, the cost of tislelizumab, and disease progression utility. At a willingness-to-pay threshold of $19,067 per QALY, TLE-CHM had an 0.8% probability of being cost-effective compared to PLB-CHM.

**Conclusion:**

From the perspective of the Chinese healthcare system, TLE-CHM is not a cost-effective first-line treatment for advanced G/GEJ adenocarcinoma compared to chemotherapy.

## 1 Introduction

Globally, gastric cancer, including gastro-oesophageal junction cancer, ranks fifth in incidence among malignant tumors, with approximately 1.1 million new cases annually (Smyth et al., 2020; Sung et al., 2021). It is also the fourth leading cause of cancer mortality, resulting in nearly 770,000 deaths each year. Notably, China bears a significant portion of this burden, accounting for 43.9% of global gastric cancer cases and 48.6% of related deaths (Morgan et al., 2022).

Despite surgery being a key treatment for gastric or gastro-oesophagogastric junction (G/GEJ) cancer, most patients present with locally advanced disease or metastases at diagnosis, precluding surgical intervention (Smyth et al., 2016) and resulting in a poor prognosis, with less than 5% surviving beyond 5 years (Zhu et al., 2023). Over 90% of G/GEJ cancers are adenocarcinomas, and approximately 80% of these patients are human epidermal growth factor receptor 2 (HER2) negative (Janjigian et al., 2021). The standard first-line treatment for HER2-negative advanced G/GEJ adenocarcinoma has been platinum-based agents plus 5-fluorouracil (Cheng et al., 2019; Lordick et al., 2022), but these have yielded unsatisfactory results, with median overall survival (OS) of less than 12 months (Al-Batran et al., 2008; Kang et al., 2009; Chen et al., 2018), making the exploration of new therapeutic approaches urgent.

Recent studies have shown that immune checkpoint inhibitors (ICIs) offer survival benefits for patients with HER2-negative advanced G/GEJ adenocarcinoma (Kang et al., 2022; Rha et al., 2023; Xu et al., 2023). A recent phase III clinical trial (RATIONALE-305) evaluated tislelizumab, an ICI, and demonstrated that tislelizumab plus chemotherapy (TLE-CHM) significantly extended the median OS to 15.0 months compared to 12.9 months with placebo plus chemotherapy (PLB-CHM), along with an improvement in median progression-free survival (PFS) (6.9 months vs. 6.2 months), in a safe and controlled manner (Qiu et al., 2024). Hence, TLE-CHM has the potential to become a first-line treatment option for HER2-negative advanced G/GEJ adenocarcinoma.

While TLE-CHM has shown promising clinical outcomes, its cost-effectiveness needs careful consideration, especially in resource-limited countries like China, given the higher costs associated with adding tislelizumab compared to PLB-CHM. Currently, no economic evaluation of TLE-CHM as a first-line treatment for HER2-negative advanced G/GEJ adenocarcinoma exists, which could impede decision-making for future use of tislelizumab. Therefore, we assessed the cost-effectiveness of TLE-CHM as a first-line treatment for HER2-negative advanced G/GEJ adenocarcinoma compared to chemotherapy alone from the perspective of the Chinese healthcare system. This study was designed and reported according to the Consolidated Health Economic Evaluation Reporting Standards 2022 (Supplementary Table A) (Husereau et al., 2022).

## 2 Methods

### 2.1 Model construction

**FIGURE 1:**
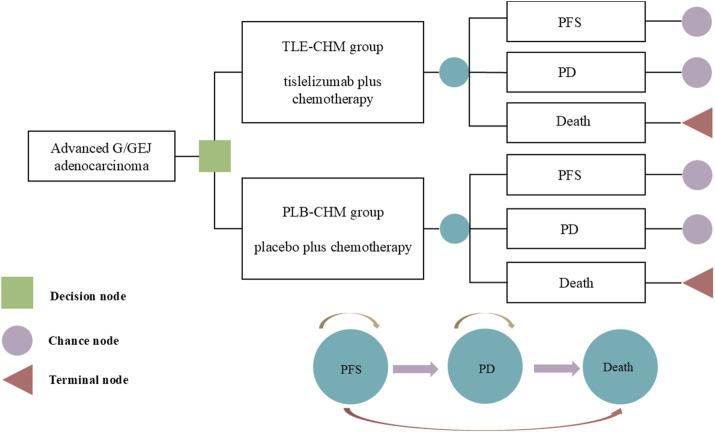
The partitioned survival model simulates outcomes based on the RATIONALE-305 trial, where all patients initially start in the PFS state and receive treatment with either TLE-CHM or PLB-CHM. As the model is run, a patient’s health status can be transformed from PFS to PD or death. G/GEJ, gastric or gastro-esophagogastric junction; PD, progressive disease; PFS, progression-free survival; PLB-CHM, placebo plus chemotherapy; TLE-CHM, tislelizumab plus chemotherapy.

A Markov partitioned survival model with three patient health states—PFS, disease progression (PD), and death—was constructed using Treeage 2025 to evaluate the cost-effectiveness of TLE-CHM versus PLB-CHM as a first-line regimen for HER2-negative advanced G/GEJ adenocarcinoma (Figure 1). It is important to note that partitioned survival models can directly use survival curves from clinical trials (e.g., OS and PFS) to partition health states. This makes them especially suitable for scenarios with limited data or where transition probabilities are hard to obtain, such as in advanced cancer research. Health states in the model are mutually exclusive. As patients progress, they either remain in their current state or transition to another without the possibility of reverting. All patients enter the model in the PFS state. The model used a cycle length of 3 weeks, with a total simulation duration of 350 cycles, which is equivalent to approximately 20 years. This duration was chosen so that by the end of the simulation, 99% of patients in both treatment groups would have died. The transition probability from PFS to death is represented by China’s background mortality rates (Compiled by the National Bureau of Statistics of China, 2024). Outcomes measured include total costs, quality-adjusted life years (QALYs), and incremental cost-effectiveness ratios (ICERs). Where ICER represents the ratio of increased cost to increased QALY for TLE-CHM compared to PLB-CHM. Based on China’s policies regarding medical insurance reimbursement and national drug price negotiations, we followed the recommendations of Cai et al. (2022) and set China’s 2023 *per capita* GDP multiplied by 1.5 ($19,067/QALY) as the willingness-to-pay (WTP) threshold. Following the Guidelines for Pharmacoeconomic Evaluation in China, a willingness-to-pay (WTP) threshold of three times China’s *per capita* GDP in 2023 ($38,133 per QALY) was applied (Yue et al., 2021). An ICER below this preset threshold indicates that the TLE-CHM regimen is cost-effective compared to PLB-CHM and *vice versa*.

### 2.2 Clinical data

Patient and clinical treatment information for this study were sourced from the RATIONALE-305 trial, a randomized, double-blind, phase 3 clinical trial (Qiu et al., 2024). Conducted across 146 medical centers in Asia, Europe, and the United States, the trial enrolled patients who were ≥18 years old, HER2-negative, had locally advanced unresectable or metastatic G/GEJ adenocarcinoma, had not received prior systemic anticancer treatment, and regardless of PD-L1 expression status. Ultimately, a total of 997 patients were enrolled in the RATIONALE-305 trial, of which 748 patients (75%) were from Asia. Specifically, 499 patients were from China, 17 were from Chinese Taiwan, 131 were from South Korea, and 101 were from Japan. Patients were randomly assigned to either the TLE-CHM or PLB-CHM group. The TLE-CHM group received tislelizumab with an investigator-selected chemotherapy regimen every 3 weeks, while the PLB-CHM group received a placebo with chemotherapy every 3 weeks. The investigator-selected chemotherapy regimens were capecitabine plus oxaliplatin or 5-fluorouracil plus cisplatin. Specifically, tislelizumab 200 mg on day 1, capecitabine 1000 mg/m^2^ twice daily on days 1–14, oxaliplatin 130 mg/m^2^ on day 1 or 5-fluorouracil 800 mg/m^2^ on days 1–5, and cisplatin 80 mg/m^2^ on day 1. Tislelizumab and capecitabine were continued until PD or intolerable toxicity, with the other drugs administered for up to six cycles. The median duration of drug treatment is shown in Supplementary Table B. Since RATIONALE-305 failed to provide detailed treatment data after the patients progressed, the best supportive care was considered as an intervention after the patients’ PD. Based on the RATIONALE-305 trial (Qiu et al., 2024) and the Chinese Gastric Cancer Treatment Guidelines (2022 edition) (National Health and Safety Commission, 2022), after PD, it is assumed that 50% of patients in the TLE-CHM group and 57% in the PLB-CHM group receive docetaxel (75 mg/m^2^ on day 1) as second-line treatment, while the remaining patients receive the best supportive care.

To obtain the probability of patients transitioning between health states, we digitized the OS and PFS survival curves for the RATIONALE-305 trial using GetData Graph Digitizer (version 1.2) software. Subsequently, we reconstructed individual patient data using R software following Guyot et al. (2012) and fitted them using a range of survival functions including exponential, gamma, gen. F, gen. Gamma, Gompertz, Weibull, log-logistic, and log-normal (Kashiwa and Matsushita, 2023). The selection of the optimal survival function was based on the Akaike Information Criterion (AIC) and Bayesian Information Criterion (BIC), i.e., the lower the AIC and BIC values, the better the fit (Supplementary Table C) (Ishak et al., 2013; Williams et al., 2017). Ultimately, we selected the log-logistic survival function (S(t)= (1+(λt)^γ^)^−1^; S: survival probability, t: time cycle, λ: scale parameter, and γ: shape parameter) to fit the PFS and OS survival curves for TLE-CHM and PLB-CHM (Table 1, Supplementary Figure A). Our simulation’s median PFS and OS closely match the RATIONALE-305 trial data. Specifically, the model-predicted median PFS for the TLE-CHM group is 7.7 months and 6.3 months for the PLB-CHM group, similar to the trial-reported 6.9 months and 6.2 months, respectively. For median OS, the model predicts 15.3 months for the TLE–CHM group and 13.0 months for the PLB-CHM group, near the trial’s reported 15.0 months and 12.9 months (Supplementary Table D). Additionally, external validation is crucial for assessing the generalizability of survival analysis models. By using external data sources—such as other clinical trials, long-term registries, or real-world studies—to verify model predictions, we can ensure their accuracy and clinical validity, thereby reducing decision-making risks associated with censored data or sample bias. In this study, to validate the extrapolation of the OS curve for PLB-CHM, we referenced long-term survival data from the CheckMate 649 Trial (Janjigian et al., 2024), which evaluated nivolumab plus chemotherapy in patients with advanced G/GEJ adenocarcinomas. The three-, four-, and 5-year survival rates for patients in the chemotherapy arm of CheckMate 649 were 19%, 10%, and 0.5%, respectively, which were broadly consistent with our extrapolated survival curve (23%, 12%, and 0.5%).

**TABLE 1:** The basic parameters of the input model and the range of sensitivity analyses.

Variable	Base value	Range		Distribution	Source
		Min	Max		
Log-logistic survival model of PFS					
PLB-CHM	Scale = 0.1597451, Shape = 1.796688	-	-	-	Qiu et al. (2024)
TLE-CHM	Scale = 0.1294224, Shape = 1.603483	-	-	-	Qiu et al. (2024)
Log-logistic survival model of OS					
PLB-CHM	Scale = 0.07671864, Shape = 1.933828	-	-	-	Qiu et al. (2024)
TLE-CHM	Scale = 0.06524044, Shape = 1.647494	-	-	-	Qiu et al. (2024)
TLE-CHM: Incidence of AEs (%)					
Decreased platelet count	11.24	8.99	13.49	Beta	Qiu et al. (2024)
Decreased neutrophil count	11.85	9.48	14.22	Beta	Qiu et al. (2024)
Anaemia	5.02	4.02	6.02	Beta	Qiu et al. (2024)
Neutropenia	6.63	5.30	7.96	Beta	Qiu et al. (2024)
PLB-CHM: Incidence of AEs (%)					
Decreased platelet count	11.54	9.23	13.85	Beta	Qiu et al. (2024)
Decreased neutrophil count	11.54	9.23	13.85	Beta	Qiu et al. (2024)
Anaemia	7.49	5.99	8.99	Beta	Qiu et al. (2024)
Neutropenia	6.88	5.50	8.26	Beta	Qiu et al. (2024)
Cost ($)					
Tislelizumab (100 mg)	177.81	142.25	213.37	Gamma	YAOZH (2024)
Capecitabine (500 mg)	0.45	0.36	0.54	Gamma	YAOZH (2024)
Oxaliplatin (100 mg)	33.20	26.56	39.84	Gamma	YAOZH (2024)
5-fluorouracil (500 mg)	7.66	6.13	9.19	Gamma	YAOZH (2024)
Cisplatin (50 mg)	10.78	8.62	12.94	Gamma	YAOZH (2024)
Docetaxel (20 mg)	8.34	6.67	10.00	Gamma	YAOZH (2024)
Decreased platelet count	1057.16	845.73	1268.59	Gamma	YAOZH (2024)
Decreased neutrophil count	83.46	66.77	100.16	Gamma	YAOZH (2024)
Anaemia	104.60	83.68	125.52	Gamma	YAOZH (2024)
Neutropenia	83.46	66.77	100.16	Gamma	YAOZH (2024)
Best supportive care per cycle	182.59	146.08	219.11	Gamma	Huang et al. (2023)
Routine follow-up per cycle	73.87	59.09	88.64	Gamma	Huang et al. (2023)
Tests per cycle	358.05	286.44	429.67	Gamma	Liu et al. (2022)
Terminal care in end-of-life	1492.49	1193.99	1790.99	Gamma	Liu et al. (2022)
Median relative dose intensity^a^					
TLE-CHM group					
Tislelizumab	100%	90.3%	100%	Beta	Qiu et al. (2024)
Oxaliplatin	100%	79.3%	99.2%	Beta	Qiu et al. (2024)
capecitabine	100%	65.4%	93.3%	Beta	Qiu et al. (2024)
5-fluorouracil/	100%	80.9%	99.2%	Beta	Qiu et al. (2024)
Cisplatin	100%	84.9%	100.2%	Beta	Qiu et al. (2024)
PLB-CHM group					
Oxaliplatin	100%	79.2%	98.6%	Beta	Qiu et al. (2024)
capecitabine	100%	65.7%	91.7%	Beta	Qiu et al. (2024)
5-fluorouracil/	100%	74.3%	94.1%	Beta	Qiu et al. (2024)
Cisplatin	100%	77.9%	97.4%	Beta	Qiu et al. (2024)
Utility value					
PFS	0.797	0.638	0.956	Beta	Shu et al. (2022)
PD	0.577	0.462	0.692	Beta	Shu et al. (2022)
Disutility due to AEs					
Decreased platelet count	−0.11	−0.09	−0.13	Beta	Tolley et al. (2013)
Decreased neutrophil count	−0.20	−0.16	−0.24	Beta	Nafees et al. (2017)
Anaemia	−0.07	−0.06	−0.08	Beta	Cai et al. (2021)
Neutropenia	−0.20	−0.16	−0.24	Beta	Nafees et al. (2017)
Body surface area (m^2^)	1.72	1.38	2.06	Normal	Huang et al. (2023)
Discount rate	0.05	0.00	0.08	Fixed	Yue et al. (2021)

^a^ Relative dose intensity is actual dose intensity/planned dose intensity; AE, adverse event; OS, overall survival; PD, progressive disease; PFS, progression-free survival; PLB-CHM, placebo plus chemotherapy; TLE-CHM, tislelizumab plus chemotherapy.

### 2.3 Cost and utility estimates

The study included only direct medical costs, encompassing expenses for medications, tests, routine follow-up, the best supportive care, management of serious adverse reactions (≥grade 3) with an incidence of ≥5%, and end-of-life care (Table 1). Drug costs were based on national bidding prices (YAOZH, 2024), while other costs were sourced from published literature and adjusted to 2023 values using the China Bureau of Statistics medical price index (Compiled by National Bureau of Statistics of China, 2024). The cost of managing adverse reactions was calculated by multiplying the cost of managing each adverse reaction by its probability of occurrence and summing these values across all adverse reactions, assuming all adverse reactions occur in the first treatment cycle. All costs were expressed in U.S. dollars at the average 2023 exchange rate (1 USD = 7.05 RMB). To facilitate the calculation of the patient’s medication dosage, we assumed the patient’s body surface area is 1.72 m^2^ (Liu et al., 2022).

Utility value is an indicator for assessing patients’ social function and overall health status, including physical, psychological, and disease-related aspects. It is measured on a scale from 0 to 1, where 0 represents death and 1 represents the best possible health, with patients’ health typically falling between these two extremes. Due to the lack of quality-of-life data in the RATIONALE-305 trial, utility values for PFS and PD were derived from published Chinese literature (Table 1). The model also considered the disutility of serious adverse reactions (≥grade 3) with an incidence of ≥5%. Specifically, the disutility of each adverse reaction is calculated by multiplying the given disutility by the adverse reaction incidence rate, and then these disutility values are subtracted from the total utility value. Both costs and utilities were discounted at an annual rate of 5% (Yue et al., 2021).

### 2.4 Sensitivity analysis

To assess the robustness of the model results, a sensitivity analysis was conducted, including one-way and probabilistic sensitivity analyses. The impact of parameter variations on the model outcomes was assessed through the one-way sensitivity analysis. In the one-way sensitivity analysis, all parameters were adjusted within their reported 95% confidence intervals. For parameters without reported confidence intervals, fluctuations of ±20% from the baseline value were assumed, with the discount rate varying between 0% and 8% (Table 1). Some patients may reduce the drug dose due to intolerance during treatment. In our model, the relative intensity of the drug dose (the ratio of the actual dose intensity to the planned dose intensity) varies based on data from the RATIONALE-305 trial. The results are presented in a tornado diagram. For the probabilistic sensitivity analysis, 1,000 Monte Carlo simulations were performed based on a specific distribution, varying random and simultaneous preset parameters (Table 1). The results are depicted by a scatter plot and cost-effectiveness acceptability curve.

### 2.5 Subgroup analyses

To assess the impact of PD-L1 tumor area positivity (TAP) scoring and race on model outcomes, we conducted an exploratory subgroup analysis targeting populations with PD-L1 TAP scores of <5% and ≥5%, as well as Asian and North American/European populations. This analysis aims to elucidate the potential influence of different scores and race backgrounds on treatment efficacy, providing more targeted insights for clinical decision-making. Due to the lack of sufficient survival data for subgroups, for subgroup survival extrapolation, we used the same survival function in all subgroups of the PLB-CHM group as in the overall population of the PLB-CHM group. Subgroup hazard ratio (HR) from the RATIONALE-305 trial were applied to calculate the ICERs for each subgroup, following the method described by Hoyle et al. (2010), and to assess the probability of cost-effectiveness acceptability. Specifically, the shape parameter in the TLE-CHM group was equivalent to that in the PLB-CHM group, and the scale parameter in the TLE-CHM group was equivalent to the scale parameter of the PLB-CHM group multiplied by the HR.

### 2.6 Scenario analysis

Two scenarios were analyzed for the overall population. In scenario 1, the model duration was varied to 5, 10, and 15 years to assess its impact on the results. Scenario 2 explored the economics of the two treatment options at different discount rates, using 3% and 8%. Scenario 3: Although the WTP threshold in this study was set at three times China’s *per capita* GDP, which is within the range recommended by the Guidelines for Pharmacoeconomic Evaluation in China (Yue et al., 2021) and commonly used in literature (Liu et al., 2025; Zhou et al., 2025; Zhu et al., 2025), Cai et al. (2022) argued that Chinese health insurance policymakers, with stronger bargaining power, tend to prefer lower thresholds. They suggested that 1.5 times the *per capita* GDP could serve as a reference threshold for health insurance decision-makers to minimize suboptimal decisions. Therefore, we adjusted the WTP threshold to 1.5 times the *per capita* GDP of China to analyze the cost-effectiveness of TLE-CHM. In scenario 3, we replace the log-logistic distribution with alternative distributions (e.g., exponential, log-normal, Weibull) to assess the robustness of model outcomes. In scenario 4, we address the dynamic uncertainty inherent in multi-state survival models by employing a Dirichlet distribution to model the probability distribution of survival states (including PFS, PD, and death) within each cycle. Robustness of the model outcomes under uncertainty is evaluated through 1,000 Monte Carlo simulations.

## 3 Results

### 3.1 Base case analysis

The study results are presented in terms of total costs, QALYs, and ICERs (Table 2). The TLE-CHM group achieved 1.53 QALYs at a cost of $23,484.39, while the PLB-CHM group achieved 1.14 QALYs at a cost of $12,123.52. The TLE-CHM group demonstrated an incremental effectiveness of 0.38 QALYs and an incremental cost of $11,361.27, resulting in an ICER of $29,608.51 per QALY gained. These findings indicate that TLE-CHM is not a cost-effective strategy for treating HER2-negative advanced G/GEJ adenocarcinoma compared to PLB-CHM, given China’s WTP threshold of $19,067 per QALY.

**TABLE 2:** The cost and outcome results of the cost-effectiveness analysis.

Regimen	Total cost ($)	Total effectiveness (QALYs)	Incremental cost ($)	Incremental effectiveness (QALYs)	ICER ($/QALY)	Cost-effectiveness probability
Overall population						
TLE-CHM group	23,484.39	1.53	11,361.27	0.38	29,608.51	0.8%
PLB-CHM group	12,123.52	1.14	-	-	-	-
Subgroup population						
PD-L1 TAP score ≥5% population						
TLE-CHM group	24,549.08	1.55	12,425.96	0.41	27,581.54	1.8%
PLB-CHM group	12,123.12	1.14	-	-	-	-
PD-L1 TAP score <5% population						
TLE-CHM group	19,153.23	1.24	7,030.11	0.10	72,348.64	0%
PLB-CHM group	12,123.12	1.14	-	-	-	-
Asian population						
TLE-CHM group	21,304.63	1.37	9,181.50	0.23	39,818.77	0%
PLB-CHM group	12,123.12	1.14	-	-	-	-

ICER, incremental cost-effectiveness ratio; PD-L1, programmed death-ligand 1; PLB-CHM, placebo plus chemotherapy; QALY, quality-adjusted life year; TAP, tumour area positivity; TLE-CHM, tislelizumab plus chemotherapy.

### 3.2 Sensitivity analysis

**FIGURE 2:**
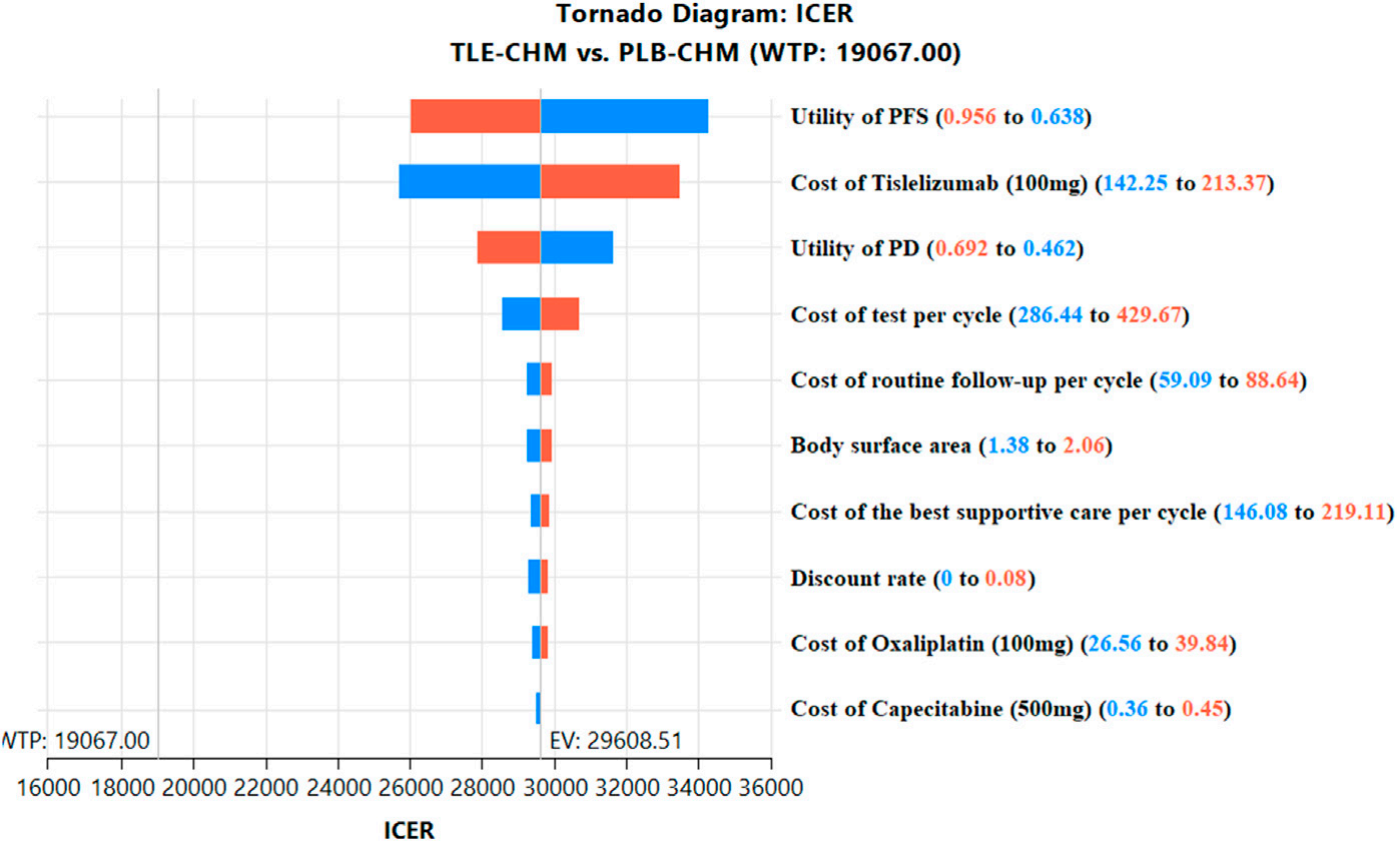
The top 10 results of one-way sensitivity analyses comparing the TLE-CHM to the PLB-CHM in the overall population. The tornado diagram visually depicts how changes in model parameters affect the ICER of the TLE-CHM treatment strategy relative to the PLB-CHM treatment strategy, with a solid line representing the predetermined WTP threshold value ($19,067/QALY). ICER, incremental cost-effectiveness ratio; PD, progressive disease; PFS, progression-free survival; PLB-CHM, placebo plus chemotherapy; TLE-CHM, tislelizumab plus chemotherapy; WTP, willingness-to-pay.

**FIGURE 3:**
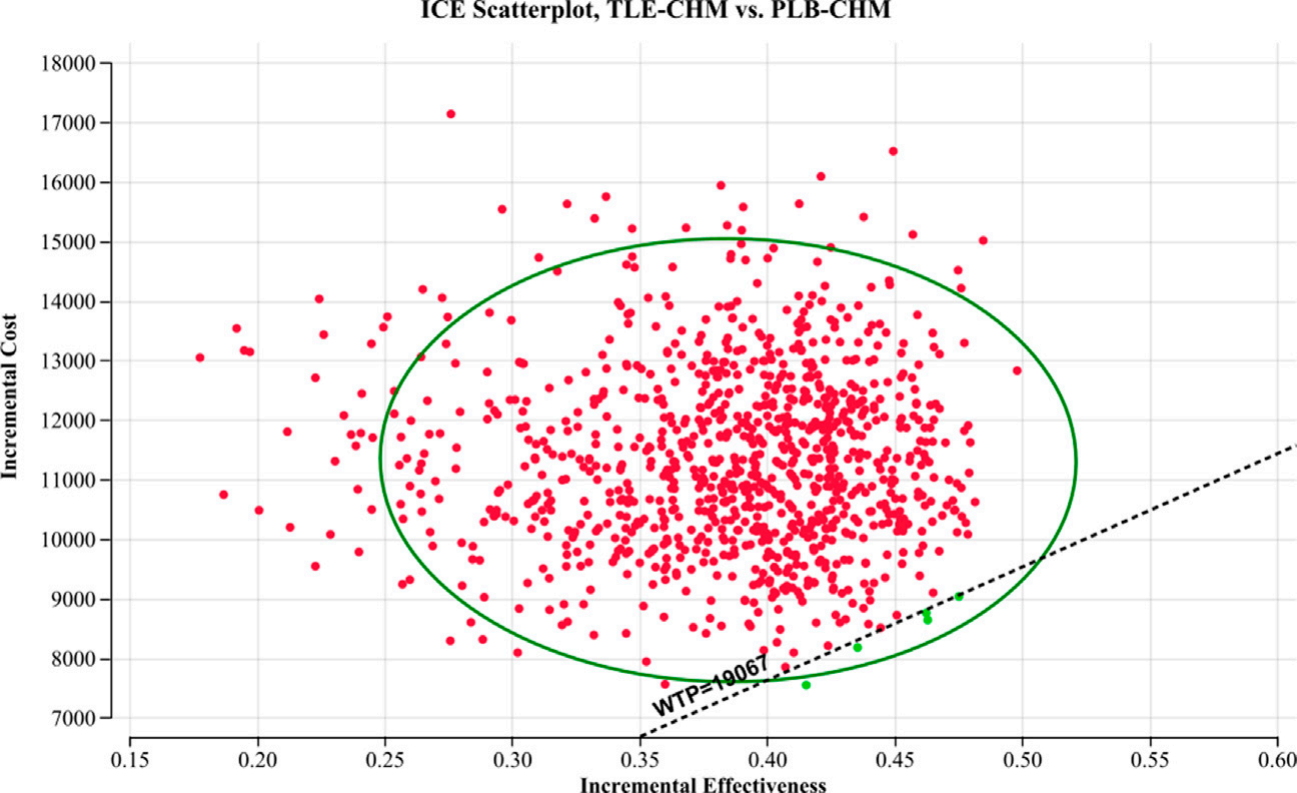
A probabilistic scatter plot of the ICER between the TLE-CHM group and the PLB-CHM group. Each point represents the ICER for one simulation. Ellipses indicate 95% confidence intervals. Simulations with points below the ICER threshold are considered cost-effective. ICE, incremental cost-effectiveness; PLB-CHM, placebo plus chemotherapy; TLE-CHM, tislelizumab plus chemotherapy; WTP: willingness-to-pay.

**FIGURE 4:**
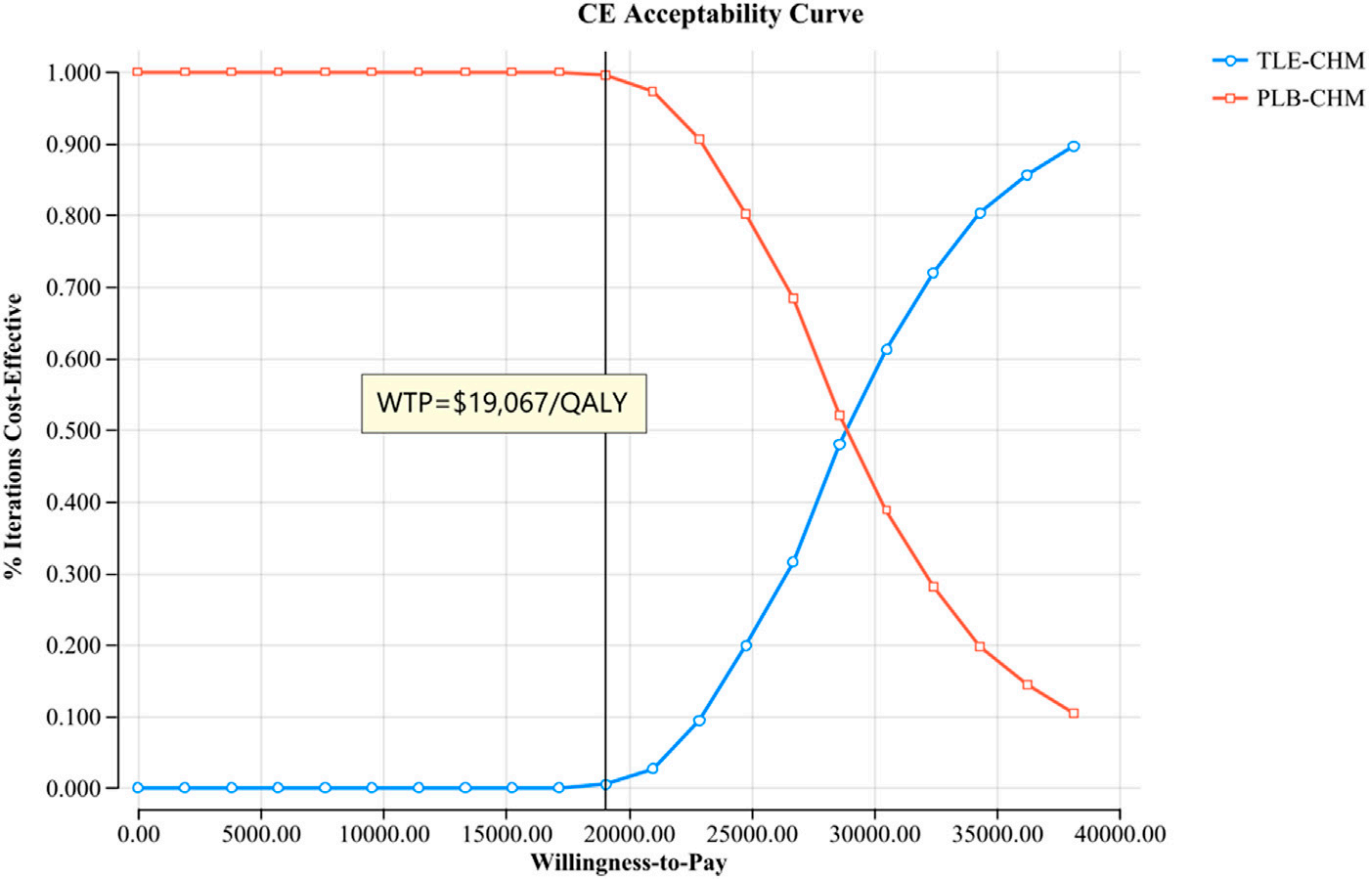
The cost-effectiveness acceptability curves for the TLE-CHM group compared to the PLB-CHM group. CE, cost-effectiveness; PLB-CHM, placebo plus chemotherapy; TLE-CHM, tislelizumab plus chemotherapy; WTP, willingness-to-pay.

The results of the one-way sensitivity analysis, presented in a tornado diagram (Figure 2), indicate that the parameters with the most significant impact on the model are the PFS utility, the cost of tislelizumab, and the PD utility. However, even with variations in these parameters, the ICER consistently remains above the predetermined WTP thresholds. This stability suggests that changes in parameter values do not affect the model’s conclusions. The probabilistic sensitivity analysis results, displayed as a scatter plot (Figure 3) and cost-effectiveness acceptability curve (Figure 4), show that at a WTP threshold of $19,067 per QALY, the probability of TLE-CHM being cost-effective compared to PLB-CHM is only 0.8%.

### 3.3 Subgroup analysis

The results of the subgroup analysis are presented in Table 2. In the subgroup with a PD-L1 TAP score ≥5%, compared to PLB-CHM, TLE-CHM increased QALYs by 0.41, with a cost of $12,425.96, resulting in an ICER of $27,581.54 per QALY gained, which is above the predefined WTP threshold, and the probability of TLE-CHM being cost-effective was 1.8%. Similarly, in the subgroup with a PD-L1 TAP score <5%, TLE-CHM only increased QALYs by 0.10, with an incremental cost of $7,030.11, leading to an ICER of $72,348.64 per QALY gained, which is substantially above the WTP threshold, with a cost-effectiveness probability of 0%. These results indicate that, compared to PLB-CHM, TLE-CHM is not cost-effective, regardless of the PD-L1 TAP score. Additionally, in the Asian population, TLE-CHM increased QALYs by 0.23 compared to PLB-CHM, with a cost of $9,181.50, resulting in an ICER of $39,818.77 per QALY gained, which is above the predefined WTP threshold. This suggests that TLE-CHM is not a cost-effective treatment option in the Asian population.

### 3.4 Scenario analysis

The scenario analysis results are presented in Table 3. In scenario 1, where the model duration varied to 5, 10, and 15 years, the ICER of TLE-CHM was $41,081.32/QALY, $33,248.53/QALY, and $30,863.74/QALY, respectively, compared to PLB-CHM. The ICER gradually decreases as the model duration increases. In scenario 2, adjusting the discount rate to 0.03 and 0.08 resulted in ICERs of $29,486.03/QALY and $29,788.05/QALY for TLE-CHM compared to PLB-CHM, respectively. In scenario 3, when the WTP threshold is adjusted to $19,067/QALY, TLE-CHM has only a 0.6% probability of being cost-effective compared to PLB-CHM. In scenario 3, when the survival distributions were adjusted to Weibull, log-normal, and exponential, the ICERs of TLE-CHM compared to PLB-CHM were $35,739.34/QALY, $29,234.05/QALY, and $37,433.23/QALY, respectively. All of these values were above the predefined WTP threshold. As shown in Supplementary Figure B, the results of scenario 4 indicate that when the Dirichlet distribution is employed to model the probability distribution of survival states within each cycle, the probability of TLE-CHM being cost-effective is 28.10%.

**TABLE 3:** Results of scenario analyses.

Scenarios	Cost ($)		QALY		ICER ($/QALY)
	TLE-CHM group	PLB-CHM group	TLE-CHM group	PLB-CHM group	
Scenario 1					
Model runtime (year) = 5	20072.53	11135.60	1.23	1.02	41081.32
Model runtime (year) = 10	22123.18	11771.89	1.41	1.10	33248.53
Model runtime (year) = 15	22974.02	11998.94	1.48	1.13	30863.74
Scenario 2					
Discount rate = 0.03	23576.10	12154.52	1.53	1.15	29486.03
Discount rate = 0.08	23352.12	12077.71	1.51	1.14	29788.05
Scenario 3					
Exponential distribution	21319.67	11324.15	1.33	1.05	35739.34
Log-normal distribution	23774.99	11782.52	1.51	1.11	29234.05
Weibull distribution	21059.97	11004.34	1.26	0.99	37433.23

ICER, incremental cost-effectiveness ratio; PLB-CHM, placebo plus chemotherapy; QALY, quality-adjusted life year; TLE-CHM, tislelizumab plus chemotherapy.

## 4 Discussion

The rising incidence of G/GEJ cancer has led to the emergence of numerous new diagnostic and therapeutic approaches in clinical practice. However, these advancements have significantly increased healthcare costs. Consequently, conducting economic evaluations of these novel strategies based on economic theories is both urgent and necessary. The RATIONALE-305 trial demonstrated that TLE-CHM, as an initial treatment, provided significant clinical benefits for patients with HER2-negative advanced G/GEJ adenocarcinoma, introducing a new first-line option. Given these positive results, TLE-CHM is expected to be widely adopted for HER2-negative G/GEJ adenocarcinoma. However, the resulting surge in economic burden will pose serious challenges for policymakers, physicians, and patients. In this context, a comprehensive cost-effectiveness analysis of TLE-CHM becomes crucial. Such evaluations have significant implications for clinical decision-making, patient outcomes, and healthcare policy. Clinicians can use this information to select the most efficient treatments, ultimately improving patient outcomes by ensuring that resources are used effectively. For patients, this means access to more effective and economically viable treatments, potentially enhancing their quality of life and reducing financial burdens. Moreover, the results have the potential to influence healthcare policy by highlighting cost-effective practices that can be adopted broadly, leading to more sustainable and equitable healthcare solutions.

This study pioneered the cost-effectiveness analysis of TLE-CHM as a first-line therapy for HER2-negative advanced G/GEJ adenocarcinoma, providing a valuable reference for both China and the international community. This constitutes the core innovation of our research. The study found that TLE-CHM costs an additional $29,608.51 per QALY compared to PLB-CHM, which is above the predetermined WTP value of $19,067/QALY. Therefore, TLE-CHM as the preferred regimen for HER2-negative advanced G/GEJ adenocarcinoma is not cost-effective within China’s healthcare system compared to chemotherapy alone. Sensitivity analysis confirmed the robustness of our results. Subgroup analyses of the PD-L1 TAP scores ≥5% and <5% show that neither subgroup achieves cost-effectiveness, aligning with the overall population results. However, the ICER for the PD-L1 TAP score ≥5% subgroup is lower than that for the overall population and the PD-L1 TAP score <5% subgroup, indicating TLE-CHM is relatively more cost-effective in the former. These findings highlight the PD-L1 TAP score’s crucial role in treatment decisions for HER2-negative advanced G/GEJ adenocarcinoma with TLE-CHM, suggesting its use to tailor treatment plans for better therapeutic and economic outcomes. Additionally, racial subgroup analyses reveal that TLE-CHM remains cost-ineffective in Asian populations, further supporting the view that TLE-CHM is not a cost-effective option for the Chinese population. Scenario 1 analysis showed that TLE-CHM becomes increasingly cost-effective with longer treatment durations, encouraging patients to adhere to the treatment as much as possible. This is favorable for doctors, patients, and families, aligning with social and ethical expectations. The Scenario 2 analysis found that the ICER values for TLE-CHM do not appear to change much when the discount rate changes. The results of Scenarios 3 and 4 both indicate that TLE-CHM is not cost-effective, further reinforcing the robustness of our model’s findings. Scenario 3 shows that when the WTP threshold is set at 1.5 times China’s *per capita* GDP, TLE-CHM has only a 0.6% probability of being cost-effective. This offers more economic insights for Chinese health insurance policymakers in setting reimbursement policies.

Currently, economic analyses of novel anti-cancer drugs as first-line treatments for advanced G/GEJ cancer are inconclusive in China. Jiang et al. (2022), Shu et al. (2022), Cao et al. (2023), and Zhang et al. (2023) found nivolumab plus chemotherapy is not cost-effective. Huang et al. (2023) and Lei et al. (2024) considered zolbetuximab plus chemotherapy unlikely to be cost-effective for CLDN18.2-positive, HER2-negative advanced G/GEJ adenocarcinoma. Lang et al. (2024) and Zheng et al. (2024) reported pembrolizumab plus chemotherapy to have lower cost-effectiveness than chemotherapy alone for advanced HER2-negative G/GEJ cancer. Li et al. (2024) found tislelizumab plus chemotherapy not cost-effective for PD-L1-positive advanced G/GEJ cancer. These findings are consistent with the results of the present study. However, Xiang et al. (2024) considered sintilimab plus chemotherapy cost-effective for first-line treatment of unresectable advanced or metastatic G/GEJ cancer. As well, to date, nine cost-effectiveness analyses of tislelizumab as a first-line regimen for cancer have been conducted from the perspective of the Chinese healthcare system. Luo et al. (2022) concluded that tislelizumab plus chemotherapy is cost-effective for advanced non-squamous non-small cell lung cancer. Similarly, studies by Lu et al. (2023), Zhou et al. (2023), Tang et al. (2024), Zheng et al. (2024a), Zheng et al. (2024b), Zheng et al. (2024c), He et al. (2024), and Liu and Shao (2024) all found tislelizumab plus chemotherapy to be a cost-effective first-line regimen for advanced esophageal squamous cell carcinoma. Additionally, Zheng et al. (2024a), Zheng et al. (2024b), Zheng et al. (2024c) and Chen et al. (2024) found it cost-effective for advanced hepatocellular carcinoma. These results align with our study’s findings.

This study has several important strengths. First, it utilized 5-year survival data from the recently published RATIONALE-305 trial, which directly compared TLE-CHM to chemotherapy alone. Second, with 75% of patients in the RATIONALE-305 trial being from Asia, the results are highly applicable to the Chinese population, a significant strength of this study. Third, the study included subgroup and scenario analyses to evaluate economic outcomes, providing valuable insights for policymakers, physicians, and patients.

This study has several limitations. First, the RATIONALE-305 trial has not yet been completed, lacking long-term survival data. Consequently, a log-logistic survival model was used to simulate survival data beyond the follow-up period, potentially introducing bias. Second, the trial did not provide detailed treatment data for patients after PD. We assumed that some patients were treated with docetaxel after PD while others received the best supportive care, an assumption that may not accurately reflect true clinical practice. Third, the trial did not offer quality-of-survival data; thus, survival utility values were sourced from published Chinese literature, potentially leading to biased results. However, sensitivity analyses demonstrated the model’s robustness. Fourth, the study only evaluated the cost-effectiveness of TLE-CHM versus chemotherapy alone, excluding other treatments like nivolumab plus chemotherapy due to the absence of head-to-head trials. Fifth, the extrapolation of TLE-CHM survival curves has not been externally validated. Currently, tislelizumab has not been approved for first-line treatment of HER2-negative advanced G/GEJ adenocarcinoma. Once approved for this indication, we will collect real-world patient survival data to conduct external validation of the extrapolation of TLE-CHM survival curves. Despite these limitations, the study offers valuable economic insights for policymakers, physicians, and patients.

## 5 Conclusion

The study results indicate that TLE-CHM is not a cost-effective first-line treatment for HER2-negative advanced G/GEJ adenocarcinoma compared to chemotherapy alone, from the perspective of the Chinese healthcare system.

## Data Availability

The original contributions presented in the study are included in the article/Supplementary Material, further inquiries can be directed to the corresponding authors.
