# Supplementary material for: Cost-effectiveness analysis of tislelizumab plus chemotherapy as first-line treatment for HER2-negative advanced gastric or gastro-oesophageal junction adenocarcinoma
Source: Front Pharmacol. 2025 May 22;16:1500729. doi: 10.3389/fphar.2025.1500729 (PMC12137247; doi:10.3389/fphar.2025.1500729)
Supplement: Supplementary file 1 [file DataSheet1.zip › Supplementary Material.docx]

Supplementary Material

**Cost-effectiveness analysis of Tislelizumab plus chemotherapy as first-line treatment for HER2-negative advanced gastric cancer or gastro-esophageal junction adenocarcinoma**

**1. Supplementary Table A. CHEERS 2022 Checklist.**

**2. Supplementary Table B. Median duration of treatment with the drug.**

**3. Supplementary Table C. Comparison of survival models.**

**4. Supplementary Figure A. Results of the survival curve fit the TLE-CHM and PLB-CHM group.**

**5. Supplementary Table D. Comparison of mPFS and mOS in the RATIONALE-305 trial with model-estimated data.**

**6. Supplementary Figure B. A probabilistic scatter plot of the ICER between the TLE-CHM group and the PLB-CHM group of the scenario 4.**

**1.** **Supplementary Table A. CHEERS 2022 Checklist.**

| **Topic** | **No.** | **Item** | **Reported** |
| --- | --- | --- | --- |
| **Title** |  |  |  |
|  | 1 | Identify the study as an economic evaluation and specify the interventions being compared. | Yes |
| **Abstract** |  |  |  |
|  | 2 | Provide a structured summary that highlights context, key methods, results, and alternative analyses. | Yes |
| **Introduction** |  |  |  |
| **Background and objectives** | 3 | Give the context for the study, the study question, and its practical relevance for decision making in policy or practice. | Yes |
| **Methods** |  |  |  |
| **Health economic analysis plan** | 4 | Indicate whether a health economic analysis plan was developed and where available. | Yes |
| **Study population** | 5 | Describe characteristics of the study population (such as age range, demographics, socioeconomic, or clinical characteristics). | Yes |
| **Setting and location** | 6 | Provide relevant contextual information that may influence findings. | Yes |
| **Comparators** | 7 | Describe the interventions or strategies being compared and why chosen. | Yes |
| **Perspective** | 8 | State the perspective(s) adopted by the study and why chosen. | Yes |
| **Time horizon** | 9 | State the time horizon for the study and why appropriate. | Yes |
| **Discount rate** | 10 | Report the discount rate(s) and reason chosen. | Yes |
| **Selection of outcomes** | 11 | Describe what outcomes were used as the measure(s) of benefit(s) and harm(s). | Yes |
| **Measurement of outcomes** | 12 | Describe how outcomes used to capture benefit(s) and harm(s) were measured. | Yes |
| **Valuation of outcomes** | 13 | Describe the population and methods used to measure and value outcomes. | Yes |
| **Measurement and valuation of resources and costs** | 14 | Describe how costs were valued. | Yes |
| **Currency, price date, and conversion** | 15 | Report the dates of the estimated resource quantities and unit costs, plus the currency and year of conversion. | Yes |
| **Rationale and description of model** | 16 | If modelling is used, describe in detail and why used. Report if the model is publicly available and where it can be accessed. | Yes |
| **Analytics and assumptions** | 17 | Describe any methods for analysing or statistically transforming data, any extrapolation methods, and approaches for validating any model used. | Yes |
| **Characterising heterogeneity** | 18 | Describe any methods used for estimating how the results of the study vary for subgroups. | Yes |
| **Characterising distributional effects** | 19 | Describe how impacts are distributed across different individuals or adjustments made to reflect priority populations. | Yes |
| **Characterising uncertainty** | 20 | Describe methods to characterise any sources of uncertainty in the analysis. | Yes |
| **Approach to engagement with patients and others affected by the study** | 21 | Describe any approaches to engage patients or service recipients, the general public, communities, or stakeholders (such as clinicians or payers) in the design of the study. | Not applicable |
| **Results** |  |  |  |
| **Study parameters** | 22 | Report all analytic inputs (such as values, ranges, references) including uncertainty or distributional assumptions. | Yes |
| **Summary of main results** | 23 | Report the mean values for the main categories of costs and outcomes of interest and summarise them in the most appropriate overall measure. | Yes |
| **Effect of uncertainty** | 24 | Describe how uncertainty about analytic judgments, inputs, or projections affect findings. Report the effect of choice of discount rate and time horizon, if applicable. | Yes |
| **Effect of engagement with patients and others affected by the study** | 25 | Report on any difference patient/service recipient, general public, community, or stakeholder involvement made to the approach or findings of the study | Not applicable |
| **Discussion** |  |  |  |
| **Study findings, limitations, generalisability, and current knowledge** | 26 | Report key findings, limitations, ethical or equity considerations not captured, and how these could affect patients, policy, or practice. | Yes |
| **Other relevant information** |  |  |  |
| **Source of funding** | 27 | Describe how the study was funded and any role of the funder in the identification, design, conduct, and reporting of the analysis | Yes |
| **Conflicts of interest** | 28 | Report authors conflicts of interest according to journal or International Committee of Medical Journal Editors requirements. | Yes |

**2. Supplementary Table B. Median duration of treatment with the drug.**

| Parameters | Median duration of treatment, months |
| --- | --- |
| TLE-CHM group |  |
| Oxaliplatin | 4.2 |
| capecitabine | 5.8 |
| 5-fluorouracil/ | 4.1 |
| Cisplatin | 4.2 |
| Tislelizumab | 5.9 |
| PLB-CHM group |  |
| Oxaliplatin | 4.2 |
| capecitabine | 5.6 |
| 5-fluorouracil/ | 4.3 |
| Cisplatin | 4.3 |

PLB-CHM, placebo plus chemotherapy; TLE-CHM, tislelizumab plus chemotherapy.

**3.** **Supplementary Table C. Comparison of survival models.**

|  | AIC | | BIC | |
| --- | --- | --- | --- | --- |
|  | TLE-CHM group | PLB-CHM group | TLE-CHM group | PLB-CHM group |
| OS |  |  |  |  |
| Exponential | 3048.690 | 3158.818 | 3052.907 | 3163.025 |
| Gamma | 3028.778 | 3105.457 | 3037.211 | 3113.871 |
| Gen.F | 3005.647 | 3084.965 | 3022.513 | 3101.792 |
| Gen.Gamma | 3013.944 | 3093.362 | 3026.593 | 3105.982 |
| Gompertz | 3050.343 | 3152.343 | 3058.776 | 3160.757 |
| Weibull | 3036.078 | 3118.616 | 3044.512 | 3127.029 |
| Log-logistic | 3005.475 | 3082.971 | 3013.908 | 3091.384 |
| Log-normal | 3014.707 | 3095.768 | 3023.140 | 3104.181 |
| PFS |  |  |  |  |
| Exponential | 2603.052 | 2586.698 | 2607.269 | 2590.904 |
| Gamma | 2602.526 | 2571.916 | 2610.959 | 2580.329 |
| Gen.F | 2557.393 | 2594.947 | 2574.260 | 2511.773 |
| Gen.Gamma | 2521.723 | 2505.485 | 2534.373 | 2518.104 |
| Gompertz | 2565.919 | 2577.113 | 2574.353 | 2585.526 |
| Weibull | 2604.907 | 2584.024 | 2613.340 | 2592.437 |
| Log-logistic | 2507.430 | 2497.300 | 2515.863 | 2505.713 |
| Log-normal | 2522.507 | 2505.451 | 2530.940 | 2513.864 |

AIC, Akaike information criterion; BIC, Bayesian information criterion; OS, overall survival; PFS, progression-free survival; PLB-CHM, placebo plus chemotherapy; TLE-CHM, tislelizumab plus chemotherapy.

**4.** **Supplementary** **Figure A. Results of the survival curve fit the TLE-CHM and PLB-CHM group.**


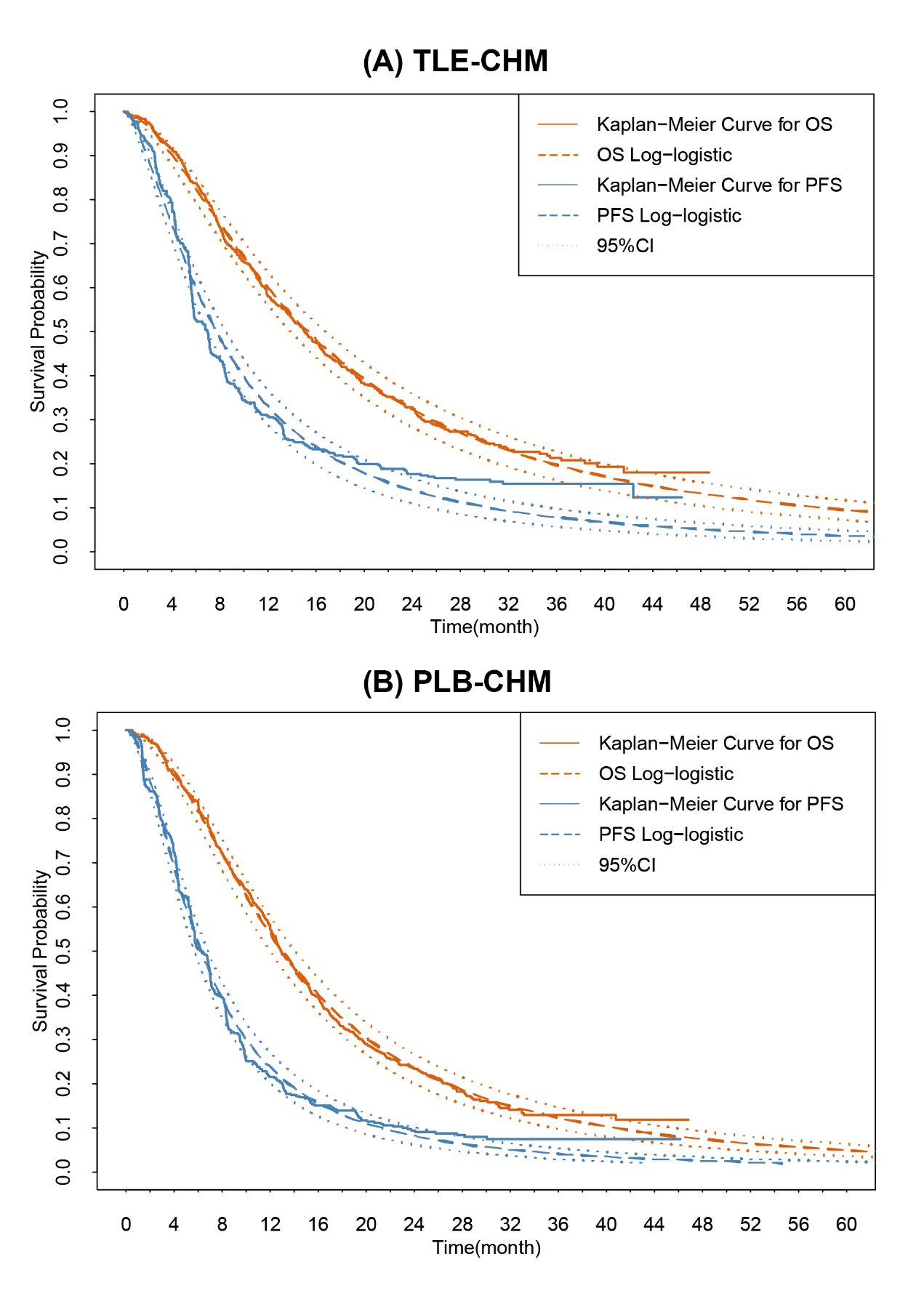


95%CI: 95% confidence interval; OS, overall survival; PFS, progression-free survival; PLB-CHM, placebo plus chemotherapy; TLE-CHM, tislelizumab plus chemotherapy.

**5.Supplementary Table D. Comparison of mPFS and mOS in the RATIONALE-305 trial with model-estimated data.**

| Treatment | | Model | RATIONALE-305 trial | Difference |
| --- | --- | --- | --- | --- |
| mPFS (mouth） | |  |  |  |
|  | TLE-CHM group | 7.7 | 6.9 | 0.8 |
|  | PLB-CHM group | 6.3 | 6.2 | 0.1 |
| mOS (mouth） | |  |  |  |
|  | TLE-CHM group | 15.3 | 15.0 | 0.3 |
|  | PLB-CHM group | 13.0 | 12.9 | 0.1 |

mPFS, median progression-free survival; mOS, median overall survival; PLB-CHM, placebo plus chemotherapy; TLE-CHM, tislelizumab plus chemotherapy.

**6.Supplementary Figure B. A probabilistic scatter plot of the ICER between the TLE-CHM group and the PLB-CHM group of the scenario 4.**


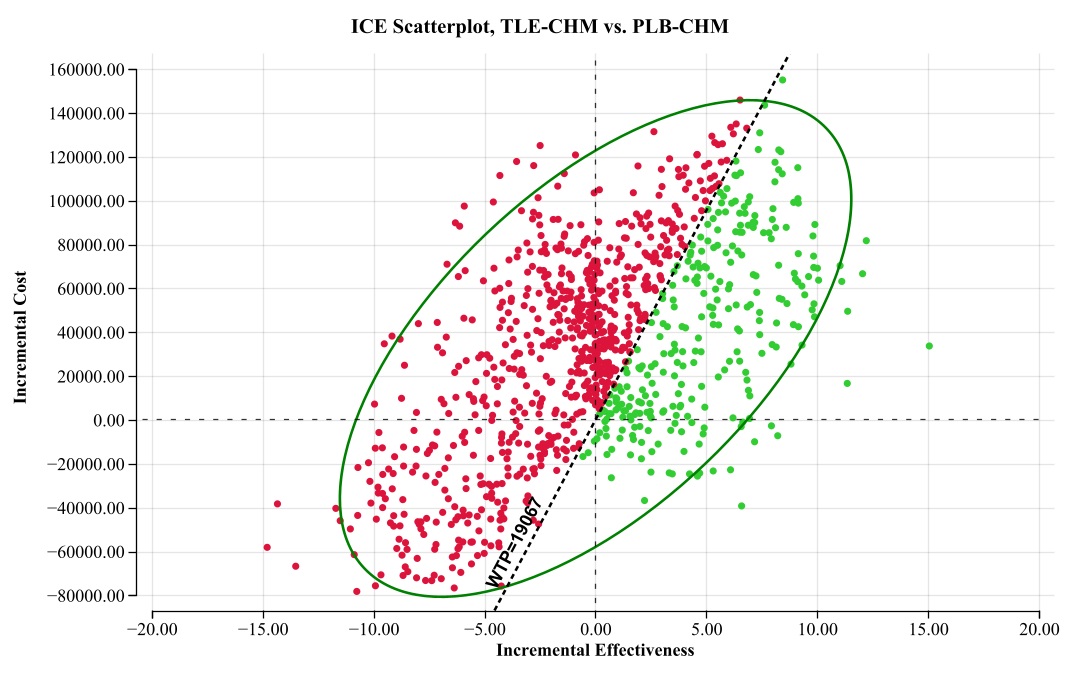


Each point represents the ICER for one simulation. Ellipses indicate 95% confidence intervals. Simulations with points below the ICER threshold are considered cost-effective. ICE, incremental cost-effectiveness; PLB-CHM, placebo plus chemotherapy; TLE-CHM, tislelizumab plus chemotherapy; WTP, willingness-to-pay.
